# Novel FDA-approved bexagliflozin drug for treating type 2 diabetes mellitus

**DOI:** 10.1097/MS9.0000000000002624

**Published:** 2024-10-07

**Authors:** Muhammad M. Saleem, Muhammad S. Zubair, Soha Abbas, Moussa Hojeij

**Affiliations:** a Dow Medical College, Dow University of Health Sciences, Karachi, Sindh, Pakistan; b Faculty of Medicine, Lebanese University, Beirut, Lebanon

## Introduction

Highlights

- Type 2 diabetes mellitus (T2DM) poses a substantial global healthcare challenge.
- SGLT2 inhibitors are a class of drugs that disrupt glucose reabsorption.
- Bexagliflozin is an FDA-approved SGLT2 inhibitor developed by Theracosbio.
- Bexagliflozin lowers HbA1c, FPG, and systolic BP in T2DM patients.
- Bexagliflozin has potential adverse effects, including ketoacidosis and UTIs.

Type 2 diabetes mellitus (T2DM) is recognized as one of the major healthcare challenges faced globally, impacting the patient’s functional capacities and quality of life, leading to significant morbidity and premature mortality^1^. It is characterized by hyperglycemia, insulin resistance, and relative impairment in insulin secretion and, if not managed properly, it can lead to severe complications such as coronary heart disease (CHD) or chronic kidney disease (CKD)^2^. Research suggests a link between T2DM and an increased risk of cardiovascular events such as in-stent restenosis in peripheral arterial disease^3^ and, modification of lipoproteins, via nitration, rendering them more atherogenic^4^. Despite the recent advancements in its treatment plans, it still remains a significant burden affecting 10.5% (536.6 million people) of the global population aged 20–79-year-old in 2021, with the numbers expected to rise up to 12.2% (783.2 million people) by 2045^5^. Moreover, global diabetes-related health expenditures were estimated at 966 billion USD in 2021 and are projected to reach 1054 billion USD by 2045^5^.

Glucose is transported across cell membranes by two mechanisms that are found at various sites in the body: the GLUTs, which involve facilitated diffusion, and SGLTs, which are a prime example of secondary active co-transport. SGLTs are symporters that transport Na+ ions down their concentration gradient and use the liberated energy to pump glucose against their concentration gradient^6^. SGLT2 is an important member of the family and it is found in proximal convoluted tubules of the kidney where it is responsible for reabsorbing Na+ and glucose back into the blood^7^. SGLT2 inhibitors form an important class of drugs that act by inhibiting the SGLT2 symporter, blocking the reabsorption of glucose and subsequently causing glycosuria while reducing blood glucose concentration. It is with regard to this that SGLT2 inhibitors have emerged as a significant therapeutic strategy for treating T2DM. Moreover, they preserve renal function while having cardiovascular benefits such as reducing blood pressure and even improving heart failure. Some of the notable drugs in this group, which are readily available for use, include canagliflozin, dapagliflozin, empagliflozin, ertugliflozin, and sotagliflozin^8–10^. In 2023, the FDA approved the use of another SGLT2 inhibitor, bexagliflozin, for treating T2DM after reviewing multiple successful clinical trials.

Bexagliflozin is manufactured and sold under the name Brenzavvy by Theracosbio. It is a subtype of SGLT2 inhibitors and is proven to alleviate hyperglycemia, due to which it recently received the green light from the FDA^11^. Its molecular formula is C24H29ClO7. It comes in the form of a 20 mg oral tablet, which is recommended to be taken once daily, in the morning with or without food. It is important to assess the patient’s renal function and volume status before administrating the drug since the patient’s eGFR shouldn’t be less than 30 ml/min/1.73 m^2^, and he/she shouldn’t be on dialysis or be hypovolemic^12^.

FDA approval for the use of bexagliflozin was based on several clinical trials in which it was either used as a monotherapy, in combination with metformin, or as an add-on to ongoing standard treatment plans including metformin, sulfonylureas, insulin, DPP4 inhibitors, or combinations of these agents^13–18^. Phase 3 studies showed a significant decrease in HbA1c and fasting plasma glucose (FPG) alongside modest reductions in systolic blood pressure (SBP) and body weight^13–18^. Some of the studies involved patients with co-existing T2DM and CKD^13^ or CHD^14^. The former study observed preserved renal function tests with a slight 0.08 mg/dl increase in serum creatinine and 2.41 ml/min/1.73 m^2^ decrease in eGFR by week 24. The latter study demonstrated positive results in reducing hospitalization for heart failure (HF) in patients who had a prior history of HF (5% in bexagliflozin arm vs. 9% in the placebo arm) alongside a decrease in all-cause mortality (3.45%) compared to the placebo arm (4.59%). Moreover, the study also noted a lower occurrence of adverse cardiac events (20.13%) in the bexagliflozin arm as opposed to the placebo arm (23.31%). Further research is required to establish bexagliflozin as a therapeutic alternative for HF. Bexagliflozin was also compared with other known drugs for treating type 2 diabetes mellitus, where it showed statistically significant (*P*<0.05) superiority to glimepiride, sitaglipitin, and dapagliflozin in lowering HbA1c, FPG, body weight, and SBP^17–19^. The results, including comparative statistics, have been summarized in Table 1.

**Table 1:** Major outcomes of clinical studies^13–19^.

	Allegretti *et al*.^13^		Halvorsen *et al*.^14^		Halvorsen *et al*.^15^		BEST trial^14^		Halvorsen *et al*.^15^		McMurray *et al*.^16^		Xie *et al*.^17^	
Clinical parameters	Placebo, *N*=155	Bexagliflozin, *N*=157	Placebo, *N*=138	Bexagliflozin, *N*=145	Placebo, *N*=72	Bexagliflozin, *N*=72 (5 mg), 72 (10 mg), 76 (20 mg)	Placebo, *N*=567	Bexagliflozin, *N*=1132	Sitaglipitin *N*=193	Bexagliflozin *N*=191	Glimepiride *N*=213	Bexagliflozin *N*=213	Dapagliflozin *N*=203	Bexagliflozin *N*=203
HbA1c (%)	−0.24	−0.61	−0.53^a^	−0.55^a^	+0.24	−0.31 (5 mg), −0.44 (10 mg), -0.56 (20 mg)	−0.37	−0.85	−0.82	−0.74	−0.66	−0.74	−1.08	−1.10
FPG (mmol/l)		−0.76	+1.50^a^	−1.58^a^	−0.11	−0.96 (5 mg), −1.16 (10 mg), −1.18 (20 mg)	+0.06	−1.33	−1.45	−1.82	−0.91	−1.30	−1.87	−1.95
Body mass (kg)		−1.61	+0.45^a^	−2.41^a^	−0.14	−1.58 (5 mg), −1.72 (10 mg), −1.89 (20 mg)	−0.38^b^	−3.03^b^	−0.81	−3.35	+0.60	−3.75	−2.22	−2.52
SBP (mmHg)		−3.8	−0.55^a^	−4.90^a^	−1.97	−4.15 (5 mg), −6.36 (10 mg), −5.77 (20 mg)	−6.87	−9.83	−1.90	−4.23	−7.18	−13.43	−6.3	−6.4

a Values shown were recorded at week 96.

b Values shown were recorded at week 48, while the rest were recorded at week 24.

1. Allegretti *et al*., 2019.^13^

2. Halvorsen *et al*., 2019.^14^

3. Halvorsen *et al*., 2020.^15^

4. McMurray *et al*., 2020.^16^

5. Halvorsen *et al*., 2019.^17^

6. Halvorsen, *et al*., 2023.^18^

7. Xie *et al*., 2024.^19^

FPG, fasting plasma glucose; SBP, systolic blood pressure.

Bexagliflozin is also sold under the name Bexacat, which is the first SGLT2 inhibitor for animals, particularly cats. Bexacat is known to improve glycemic control in cats. In a trial conducted by Benedict *et al*.^20^, five poorly regulated diabetic cats were administered Bexacat for over 4 weeks. Results showed a statistically significant reduction in insulin doses given to each cat, with discontinuation for two cats (*P*=0.015) alongside a decrease in FPG obtained from blood glucose concentration curves (*P*=0.022). Furthermore, adverse effects were mild with no episodes of hypoglycemia.

Keeping its beneficial actions aside, it’s important to consider and be precautious regarding bexagliflozin’s harmful effects as well. An important and common adverse event is that of ketoacidosis, a grave life-threatening condition warranting immediate hospitalization. Patients with bexagliflozin-induced ketoacidosis will display the same signs and symptoms as that of dehydration and metabolic acidosis even at low blood glucose levels (less than 250 mg/dl)^12^. Bexagliflozin-treated patients may also suffer from lower limb-related disorders such as infections, gangrene, osteomyelitis, or ischemia precipitating into the need for a lower limb amputation as observed in one of the trials (8.3 events in bexagliflozin group versus 5.1 events in placebo group per 1000 patient-years)^16^. Furthermore, bexagliflozin may also cause intravascular volume contraction, which often manifests as hypotension and plasma creatinine changes or, in a worst-case scenario, leads to acute kidney injury. Reports of serious urinary tract infections such as urosepsis and pylonephritis, genital fungal infections or Fournier’s gangrene, which is a rare but life-threatening necrotizing infection of the perineum, have been identified in the clinical trials. These conditions require urgent hospitalization, and in the case of Fournier’s gangrene, multiple surgeries or it might prove to be fatal. Lastly, bexagliflozin, when used in combination with insulin or incretins, is known to cause episodes of hypoglycemia. Thus, the FDA has recommended that the dosage of anti-hyperglycemic agents, including insulin or sulfonylureas, be reduced when using bexagliflozin, and that the patient be regularly monitored for symptoms of hypoglycemia^12^. Incidence rates of different adverse events, as reported in aforementioned trials^13–19^, have been summarized in Table 2.

**Table 2:** Adverse events of clinical studies^13–19^.

	Allegreti *et al*.^13^		Halvorsen *et al*.^14^		Halvorsen *et al*.^15^		BEST trail^16^		Halvorsen *et al*.^17^		Halvorsen *et al*.^18^		Xie *et al*.^19^	
Adverse events	Placebo, *N*=155	Bexagliflozi, *N*=157	Placebo, *N*=138	Bexagliflozin, *N*=145	Placebo, *N*=72	Bexagliflozin, *N*=72 (5 mg), 72 (10 mg), 76 (20 mg)	Placebo, *N*=567	Bexagliflozin, *N*=1132	Sitaglipitin, *N*=193	Bexagliflozin, *N*=191	Glimepiride, *N*=213	Bexagliflozin, *N*=213	Dapagliflozin, *N*=203	Bexagliflozin, *N*=203
Total affected patients, *n* (%)	105 (67.7)	109 (69.4)	94 (66.7)	93 (64.1)	29 (40.3)	26 (36.1) (5 mg), 31 (43.1) (10 mg), 36 (47.4) (20 mg)	594 (104.8)^a^	1112 (98.2)^a^	197 (102.1)^a^	180 (94.2)^a^	173 (81.2)	172 (80.8)	132 (65.0)	127 (62.6)
Serious adverse events, *n* (%)	9 (5.8)	11 (7)	12 (8.5)	4 (2.8)			26 (4.6)	373 (33)	4 (2.1)	7 (3.7)			7 (3.5)	9 (4.4)
Lower limb amputations, *n* (%)	0 (0)	1 (0.6)												
Hypovolemia or hypotension, *n* (%)	5 (3.2)	6 (3.8)												
Hypoglycemia, *n* (%)	38 (24.5)	39 (24.8)	25 (17.7)	24 (16.6)	1 (1.4)	4 (5.3)			10 (5.2)	6 (3.1)	71 (33.3)	36 (16.9)	8 (3.9)	7 (3.5)
Hyperlipidemia, *n* (%)													17 (8.4)	19 (9.4)
Hypertriglyceridemia, *n* (%)													3 (1.5)	6 (3.0)
Hyperuricemia, *n* (%)													11 (5.4)	12 (5.9)
Diabetic ketosis, *n* (%)													10 (4.9)	10 (4.9)
Renal disorders, *n* (%)	11 (7.1)	26 (16.6)	3 (2.1)	6 (4.1)	1 (1.4)	2 (2.8) (5 mg), 2 (2.8) (10 mg), 8 (10.5) (20 mg)			1 (0.5)	8 (4.2)			1 (0.5)	4 (2.0)
Urinary tract infections, *n* (%)	5 (3.2)	11 (7.0)	29 (20.6)	21 (14.5)	1 (1.4)	2 (2.8) (5 mg), 1 (1.4) (10 mg), 1 (1.3) (20 mg)			4 (2.1)	7 (3.7)	10 (4.7)	25 (11.7)	16 (7.9)	13 (6.4)
Genital mycotic infections, *n* (%)	0 (0)	5 (3.2)	2 (1.4)	2 (1.4)					0 (0)	5 (2.6)				
Musculoskeletal disorders, *n* (%)	6 (3.9)	7 (4.5)			3 (4.2)	2 (2.8) (5 mg), 4 (5.6) (10 mg), 2 (2.6) (20 mg)			3 (1.6)	5 (2.6)	20 (9.4)	18 (8.5)		
Other disorders (neurological, gastrointestinal, cardiovascular, respiratory, metabolic), *n* (%)	7 (4.5)	7 (4.5)			12 (16.7)	15 (20.8) (5 mg), 13 (18.1) (10 mg), 11 (14.5) (20 mg)			35 (18.1)	18 (9.4)	62 (29.1)	44 (20.7)	35 (17.2)	27 (13.3)

^a^ These values represent total adverse events rather than patients affected with adverse events.

1. Allegretti *et al*., 2019.^13^

2. Halvorsen *et al*., 2019.^14^

3. Halvorsen *et al*., 2020.^15^

4. McMurray *et al*., 2020.^16^

5. Halvorsen *et al*., 2019.^17^

6. Halvorsen, *et al*., 2023.^18^

7. Xie *et al*., 2024.^19^

To summarize, type 2 diabetes mellitus remains a significant challenge globally despite the recent advancements in its treatment plans. SGLT2 inhibitors have been recognized as an important potent solution to this disease. Bexagliflozin has been recently approved by the FDA after it yielded promising results in countering hyperglycemia. Despite the positive results seen in the limited number of trials, additional research is necessary to comprehensively assess bexagliflozin’s efficacy and potential side effects. For instance, comparative studies evaluating bexagliflozin’s effectiveness in comparison to other known SGLT2 inhibitors, as well as its use in patients with CHD or CKD, are some areas that require further investigation due to lack of substantial evidence. These areas could be of great interest to researchers in the future.

## Ethical approval

Ethics approval was not required for this editorial.

## Consent

Informed consent was not required for this editorial.

## Source of funding

None.

## Author contribution

M.M.S.: conceptualization, project administration, resources, supervision, validation, and writing – original draft; M.S.Z.: conceptualization, visualization, writing – original draft, and supervision; S.A.: validation, writing – review and editing, writing – original draft, resources, and visualization; M.H.: validation, writing – review and editing, resources, and visualization.

## Conflicts of interest disclosure

The authors declare no conflicts of interest.

## Research registration unique identifying number (UIN)

Not applicable.

## Guarantor

Muhammad Meeran Saleem.

## Data availability statement

Not invited.
